# Critical comparison of sample preparation strategies for shotgun proteomic analysis of formalin-fixed, paraffin-embedded samples: insights from liver tissue

**DOI:** 10.1186/1559-0275-11-28

**Published:** 2014-07-08

**Authors:** Alessandro Tanca, Marcello Abbondio, Salvatore Pisanu, Daniela Pagnozzi, Sergio Uzzau, Maria Filippa Addis

**Affiliations:** 1 Porto Conte Ricerche, S.P. 55 Porto Conte/Capo Caccia Km 8.400, Tramariglio, 07041 Alghero, Italy; 2 Dipartimento di Scienze Biomediche, Università di Sassari, Viale San Pietro 43/B, 07100, Sassari, Italy

**Keywords:** Archival tissues, FASP, FFPE, LC-MS/MS, Protein extraction

## Abstract

**Background:**

The growing field of formalin-fixed paraffin-embedded (FFPE) tissue proteomics holds promise for improving translational research. Direct tissue trypsinization (DT) and protein extraction followed by in solution digestion (ISD) or filter-aided sample preparation (FASP) are the most common workflows for shotgun analysis of FFPE samples, but a critical comparison of the different methods is currently lacking.

**Experimental design:**

DT, FASP and ISD workflows were compared by subjecting to the same label-free quantitative approach three independent technical replicates of each method applied to FFPE liver tissue. Data were evaluated in terms of method reproducibility and protein/peptide distribution according to localization, MW, pI and hydrophobicity.

**Results:**

DT showed lower reproducibility, good preservation of high-MW proteins, a general bias towards hydrophilic and acidic proteins, much lower keratin contamination, as well as higher abundance of non-tryptic peptides. Conversely, FASP and ISD proteomes were depleted in high-MW proteins and enriched in hydrophobic and membrane proteins; FASP provided higher identification yields, while ISD exhibited higher reproducibility.

**Conclusions:**

These results highlight that diverse sample preparation strategies provide significantly different proteomic information, and present typical biases that should be taken into account when dealing with FFPE samples. When a sufficient amount of tissue is available, the complementary use of different methods is suggested to increase proteome coverage and depth.

## Background

The recent development of methods suitable for analyzing the proteome of formalin-fixed, paraffin-embedded (FFPE) tissue samples can rightly be considered as one of the most promising innovations in the field of clinical proteomics [1]. Archival tissue repositories are indeed a valuable resource for protein biomarker discovery and validation, since they hold a considerable number and variety of tissue specimens, including those from patients with rare malignancies, and provide retrospective information concerning diagnosis, survival, and response to therapy [2,3].

In order to exploit this enormous potential, in the last years researchers have been aiming at evaluating the suitability of a wide array of techniques for FFPE tissue proteome analysis, including gel-based approaches (2DE-MS, DIGE-MS, GeLC-MS/MS [3-7]), shotgun LC-MS/MS (both label-based and label-free [8-13]), as well as targeted MS [14] and imaging MS [15]. With respect to shotgun LC-MS/MS, direct tissue trypsinization (DT) and protein extraction followed by in solution digestion (ISD) are the most commonly used strategies; more recently, an efficient alternative for processing FFPE protein extracts through the filter-aided sample preparation (FASP) workflow has been introduced [3,8,11]. These sample preparation workflows are all preceded by tissue deparaffinization and rehydration and usually comprise a high temperature treatment, but exhibit several differences in the other steps. Specifically, the DT workflow entails an initial tissue homogenization, which can be performed using different buffers, such as the commercial Liquid Tissue [16], as well as mixtures of ammonium bicarbonate (ABC) and acetonitrile (ACN) [17,18] or ABC and trifluoroethanol [19,20], followed by direct trypsin digestion of the tissue homogenate. The ISD workflow is instead based on a preliminary protein extraction step, comprising tissue lysis in a detergent-based buffer (usually containing SDS) and collection of soluble proteins after centrifugation, succeeded by detergent depletion (by means of dilution with ABC [21,22], dialysis [23], spin columns [24] or protein precipitation with various protocols [25-28]) and in solution digestion of the protein extract. The FASP protocol, applied with remarkable results to FFPE samples by Wiśniewski and colleagues [29-32] and recently employed with modifications by other research groups [33,34], shares the initial protein extraction step with the ISD workflow but differs in that detergent removal and protein digestion are both performed on a molecular weight cut-off centrifugal filter [35,36], instead of in solution. In spite of the growing number of FFPE tissue proteomics papers, there is currently no consensus on the optimal protocol for shotgun proteomic analysis of FFPE tissue samples, and no studies critically comparing the performance of DT, ISD and/or FASP with FFPE specimens have been reported so far [3,8,11].

In keeping with this, this work aimed to critically compare the performance of DT, FASP and ISD as sample preparation methods for shotgun proteomic analysis of FFPE tissue samples. Liver tissue was chosen as a model in consideration of its high proteome complexity in terms of expressed proteins and metabolic pathways. In addition, in consideration of organ size and morphology, variations among serial tissue slices are kept to a minimum. Consecutive microtome sections were therefore obtained from an FFPE liver sample retrieved from a hospital tissue repository and divided into nine aliquots, with the aim of performing three independent technical replicates per method. The peptide mixtures obtained with the three methods were subjected to single run, long gradient LC coupled with high resolution MS, and the MS results were analyzed according to a spectral counting approach for label-free quantification. Data were comparatively evaluated in terms of method reproducibility, as well as of differential distribution of protein/peptide identifications according to subcellular localization, MW, pI and hydrophobicity. Finally, the same MS data were re-analyzed with the purpose of evaluating the impact of non-tryptic and formaldehyde-modified peptides on DT, FASP and ISD workflows.

## Results

### Comparative evaluation of method reproducibility

**Figure 1:**
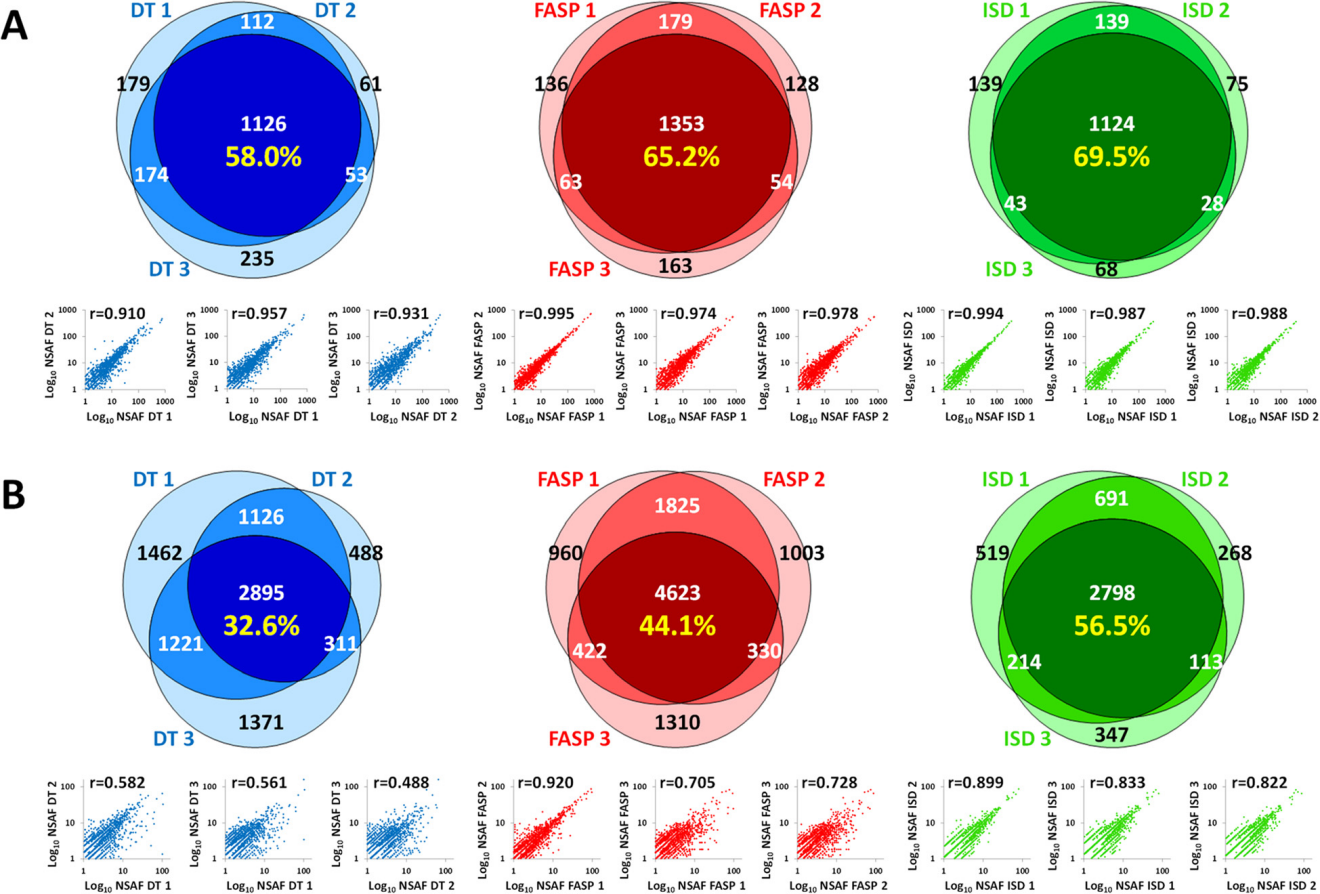
**Qualitative and quantitative reproducibility of DT (blue), FASP (red) and ISD (green) methods. A)** Top: Venn diagrams depicting distribution of identified proteins among replicates. Percentages of common proteins are indicated in yellow. Bottom: correlation of protein abundance between all replicates combinations for every method. Pearson correlation coefficients are also reported. **B)** Same as Panel A but at the peptide level.

As illustrated in Figure 1, reproducibility was assessed for each method both at protein (panel A) and peptide (panel B) level. Qualitative reproducibility was measured in terms of identification overlap (Venn diagrams), whose value was worked out based on the percentage of proteins or peptide sequences identified in all replicates, independently from their abundance. Quantitative reproducibility was instead expressed as Pearson correlation coefficients (r, dot plots), which were calculated based on the abundance values obtained for each protein or peptide in two different replicates, for a total of three replicate combinations per method. On the whole, ISD showed the highest reproducibility at all levels, followed in all cases by FASP and DT. Specifically, ISD protein identification overlap was about 70% (57% at peptide level), and the average r value was 0.990 for proteins and 0.851 for peptides; the corresponding values were 65% (44%), 0.982 and 0.784 for FASP and 58% (33%), 0.933 and 0.544 for DT. However, in terms of mean number of identifications per replicate (as detailed in Additional file 1), FASP outperformed the other methods, with 1693 proteins and 7432 peptides, against 1510 proteins and 5774 peptides for DT and 1358 proteins and 3855 peptides for ISD.

### Quantitative method comparison according to protein/peptide identification data, subcellular localization, MW, pI and hydrophobicity

**Figure 2:**
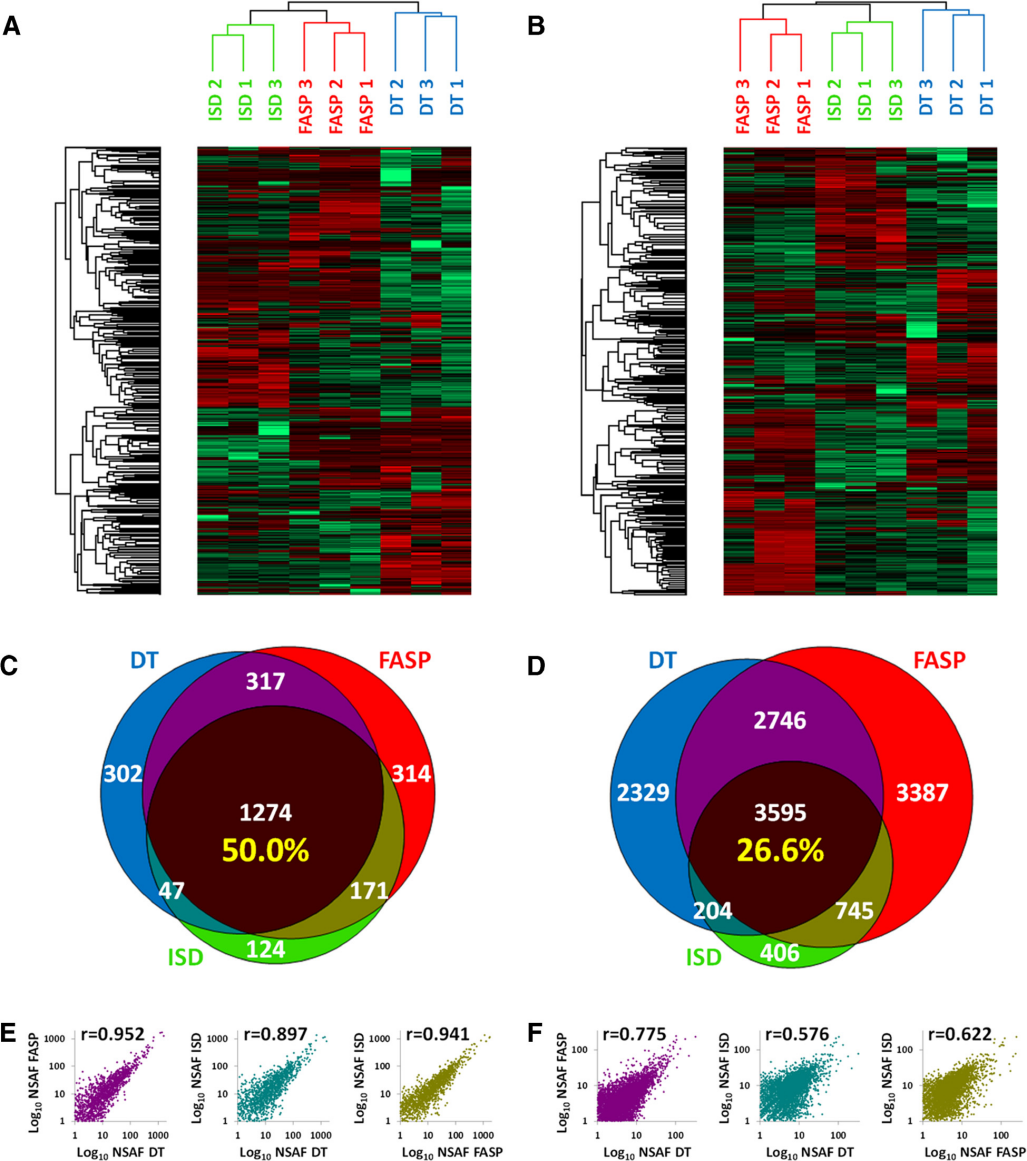
**Qualitative and quantitative method comparison.** Top: Unsupervised hierarchical cluster analysis based on protein **(A)** and peptide **(B)** label-free quantitative data, respectively. Middle: Venn diagrams illustrating distribution of all identified proteins **(C)** and peptides **(D)**. Percentage of common proteins and peptides are indicated in yellow. Bottom: Dot plots describing correlation of protein **(E)** and peptide **(F)** abundance between DT and FASP (purple), DT and ISD (blue-green), FASP and ISD (bronze). Pearson correlation coefficients are also reported.

Label-free quantitative data obtained applying DT, FASP and ISD workflows on the same FFPE tissue specimen were then parsed in order to compare the performance of each method according to several parameters and uncover their specific biases, advantages and drawbacks. First, protein and peptide abundance data were used to perform an unsupervised hierarchical cluster analysis. As shown in Figure 2A (proteins) and 2B (peptides), the three replicates of each method clustered together, consistently with a higher similarity among replicates of the same method than among different methods; moreover, when grouping samples into two clusters, DT data clustered separately from FASP-ISD both at protein and peptide level. Then, data from the three replicates of each method were merged in order to explore the overlap among the whole DT, FASP and ISD proteomes. Venn diagrams in Figure 2C and 2D illustrate overlap of protein and peptide identifications, respectively, among the three methods. Out of 2549 proteins, 50% were common to all methods, where 12%, 12% and 5% were unique for DT, FASP and ISD, respectively. Going down to the peptide level, about 27% of the 13412 peptides identified in total were common to all methods, where 17%, 25% and 3% were unique for DT, FASP and ISD, respectively. Correlation of protein or peptide abundance data between different methods was also evaluated by means of the Pearson’s r value. As shown in Figure 2E (proteins) and 2F (peptides), the highest correlation could be observed between DT and FASP both for proteins (0.952) and peptides (0.775), followed by the pairs FASP-ISD and DT-ISD.

**Figure 3:**
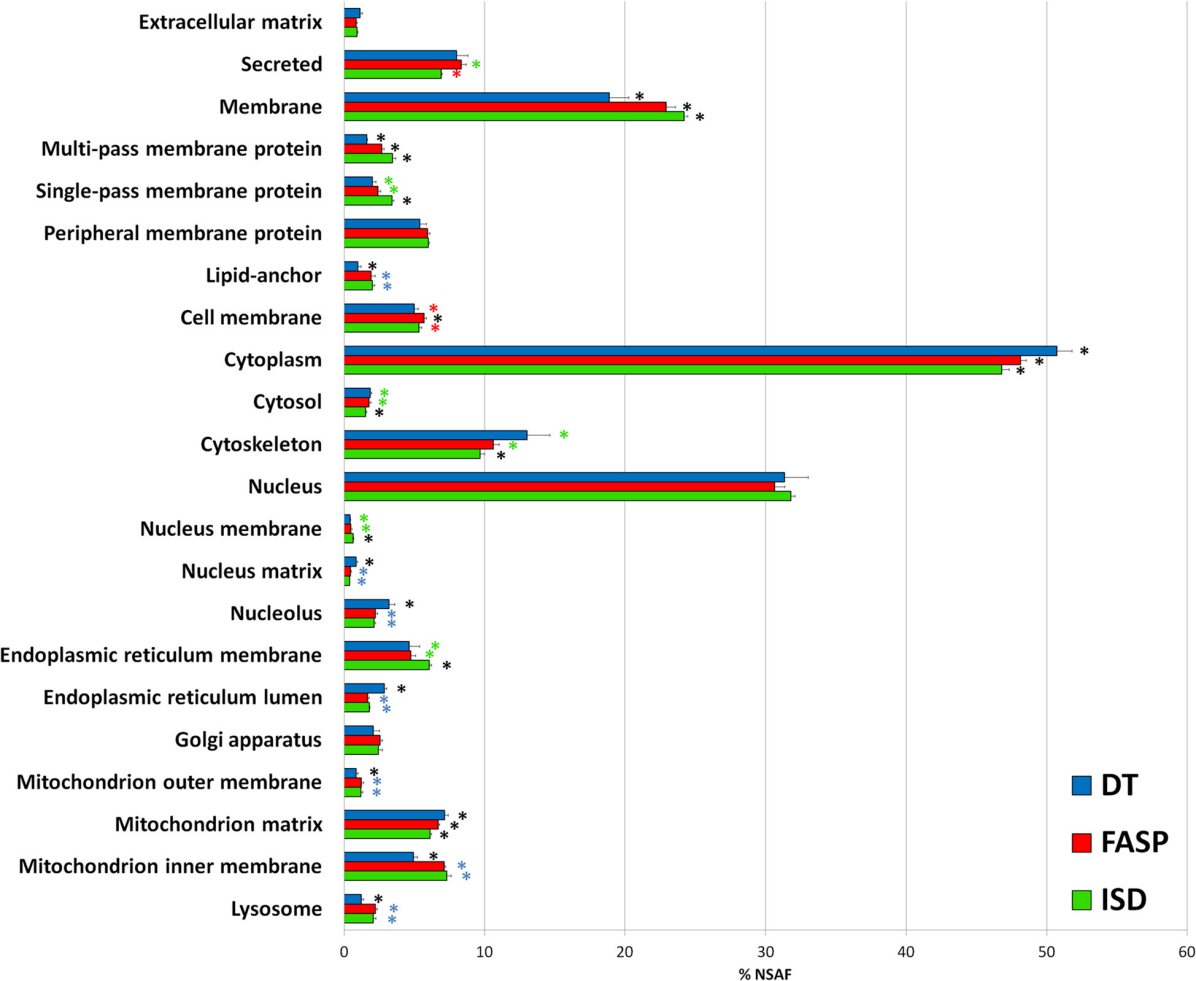
**Quantitative protein distribution according to subcellular localization.** Mean and SD value of NSAF percentage for three independent experimental replicates are shown. NSAF values were expressed as percentage of the annotated proteins. Asterisks indicate statistical significance according to Student’s t-test (p value < 0.05); the blue ones indicate statistically significant difference versus DT, the red ones versus FASP, the green ones versus ISD and the black ones versus all other methods, respectively.

One of the key aims of this study consisted in investigating the distribution of protein identifications according to their subcellular localization when using DT, FASP or ISD, in order to reveal possible biases typical of each method. Figure 3 shows the quantitative distribution of proteins according to their localization. Notwithstanding a general coherence of the three methods, slight but statistically significant differences could be observed for several specific localizations. In fact, the DT proteome was relatively depleted in membrane proteins (both considering a general “membrane” annotation or even specific locations, such as cell, nucleus, ER, and mitochondrion membranes) and enriched in proteins belonging to cytoskeleton, nucleolus, ER lumen and mitochondrial matrix, while the ISD proteome exhibited an opposite trend (higher abundance of membrane proteins and lower abundance of cytoplasmic proteins); FASP values were in most cases in an intermediate position between those of the other methods. Comparable trends (except for a slightly higher extent of membrane proteins for FASP; Additional file 2) could also be seen when considering protein percentage (each protein “weighs” one) instead of abundance (each protein “weighs” based on its NSAF value).

**Figure 4:**
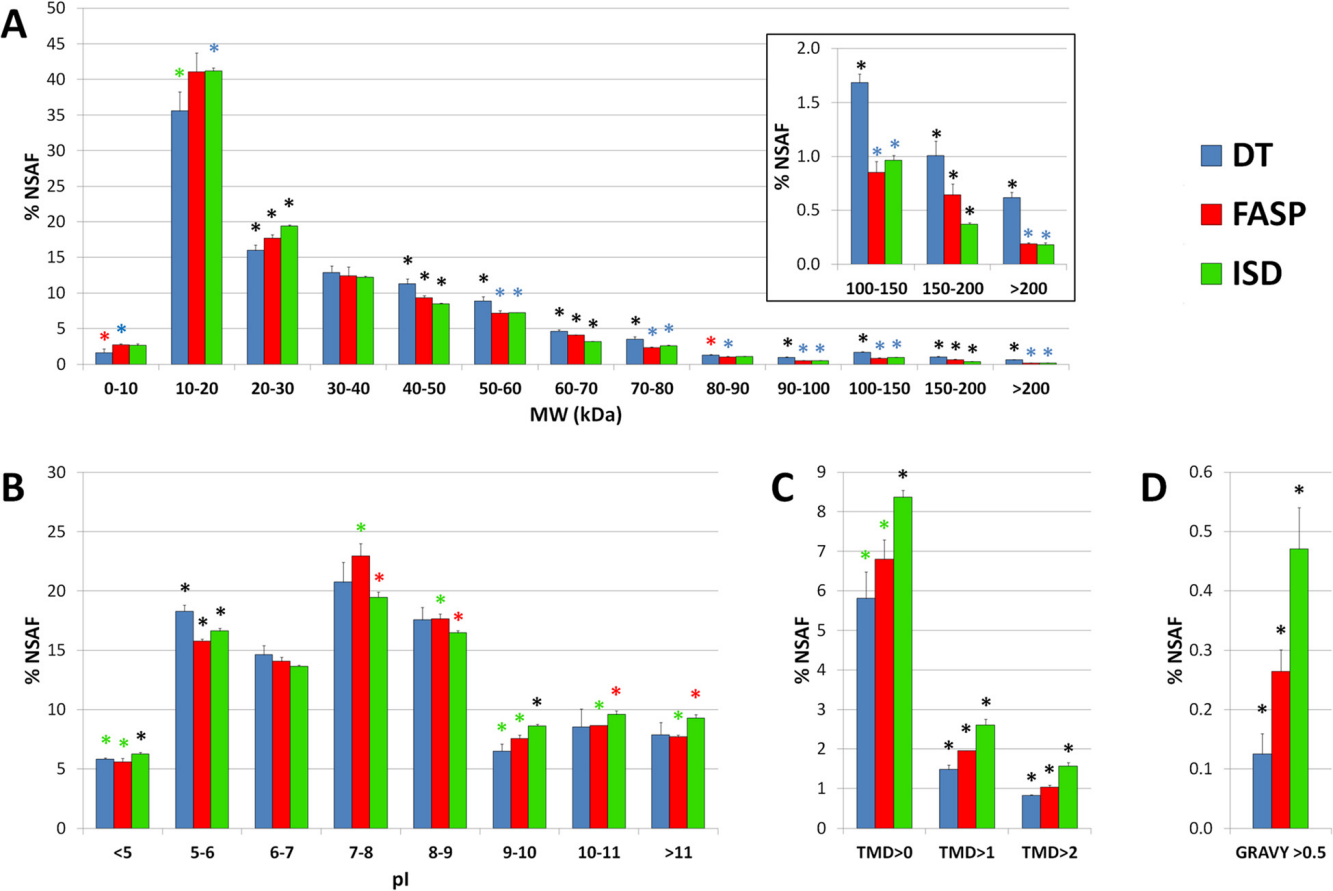
**Quantitative protein distribution according to physicochemical features.** Quantitative protein distribution according to MW **(A)**, pI **(B)**, number of transmembrane domains (TMD, **C**) and hydrophobicity (GRAVY score, **D**). Mean and SD value of NSAF percentage for three independent experimental replicates are shown. NSAF values were expressed as percentage of all proteins. Asterisks indicate statistical significance according to Student’s t-test (p value < 0.05); the blue ones indicate statistically significant difference versus DT, the red ones versus FASP, the green ones versus ISD and the black ones versus all other methods, respectively.

Protein distribution according to MW and pI was also assessed. Concerning MW, label-free quantitative proteomic data achieved for the DT samples showed a clearly different behavior when compared to FASP and ISD (Figure 4A). On the whole, proteins with MW > 40 kDa were significantly more abundant in DT, while proteins with MW < 30 kDa exhibited an opposite trend; specifically, proteins with MW comprised between 100 and 200 kDa were twice as abundant in DT as in the other methods, and those weighing more than 200 kDa were even three-fold more abundant (Figure 4A, right box). Similar data were obtained when analyzing protein percentage distribution, independently from abundance (Additional file 3A). Moreover, the mean MW of the proteome dataset was 58.9 (±1.3) for DT, 51.8 (±1.2) for FASP and 49.6 (±0.8) for ISD, with a statistically significant difference between DT and FASP (p = 0.002) as well as between DT and ISD (p = 0.0004). Significant differences could also be observed concerning pI. As illustrated in Figure 4B, the relative maximum of protein abundance was observed around acidic pIs for DT, neutral pIs for FASP and basic pIs for ISD. Moreover, the mean pI of the proteome dataset was 7.15 (±0.01) for DT, 7.24 (±0.01) for FASP and 7.24 (±0.005) for ISD, with a statistically significant difference between DT and FASP (p = 0.0005) as well as between DT and ISD (p = 0.0002).

In addition, we were interested in estimating the amount and abundance of hydrophobic and transmembrane domain (TMD) containing proteins detected with the three methods. To this aim, we measured the relative abundance of proteins predicted to have at least 1, 2 or 3 TMDs, as well as that of proteins with GRAVY score higher than 0.5 (usually considered as hydrophobic [37]). According to both results, the ISD proteome dataset was found to contain a higher relative abundance of hydrophobic and TMD-containing proteins, followed in order by FASP and DT (Figure 4C-D). However, FASP reached and exceeded ISD results, in terms of protein percentage in absolute protein identifications, respectively (Additional file 3C-D).

Proteomic data were further analyzed at a single protein level (as detailed in Additional file 4), in order to identify proteins consistently showing a differential abundance among methods. A total of 176 proteins were found to be significantly differentially abundant between one method and the remaining two; most of them (100) were different between DT and FASP/ISD, thus confirming indications previously gathered from hierarchical clustering data. Proteins enriched in DT samples (42 with p < 0.05) were mainly high-MW proteins (113 kDa on average) localized in cytoskeleton, ER and nucleus, including protein disulfide-isomerases, spectrin subunits, myosins, plectin; proteins more abundant in FASP samples (29 with p < 0.05) were mostly mitochondrial proteins, such as NADH dehydrogenase subunits; ISD proteomes showed a relative increase (23 proteins with p < 0.05) in proteins from organelle and cell membranes, as peroxisomal membrane proteins, CD67 antigen and protein kish-A.

### Assessment of the impact of non-tryptic and formaldehyde-modified peptides

**Figure 5:**
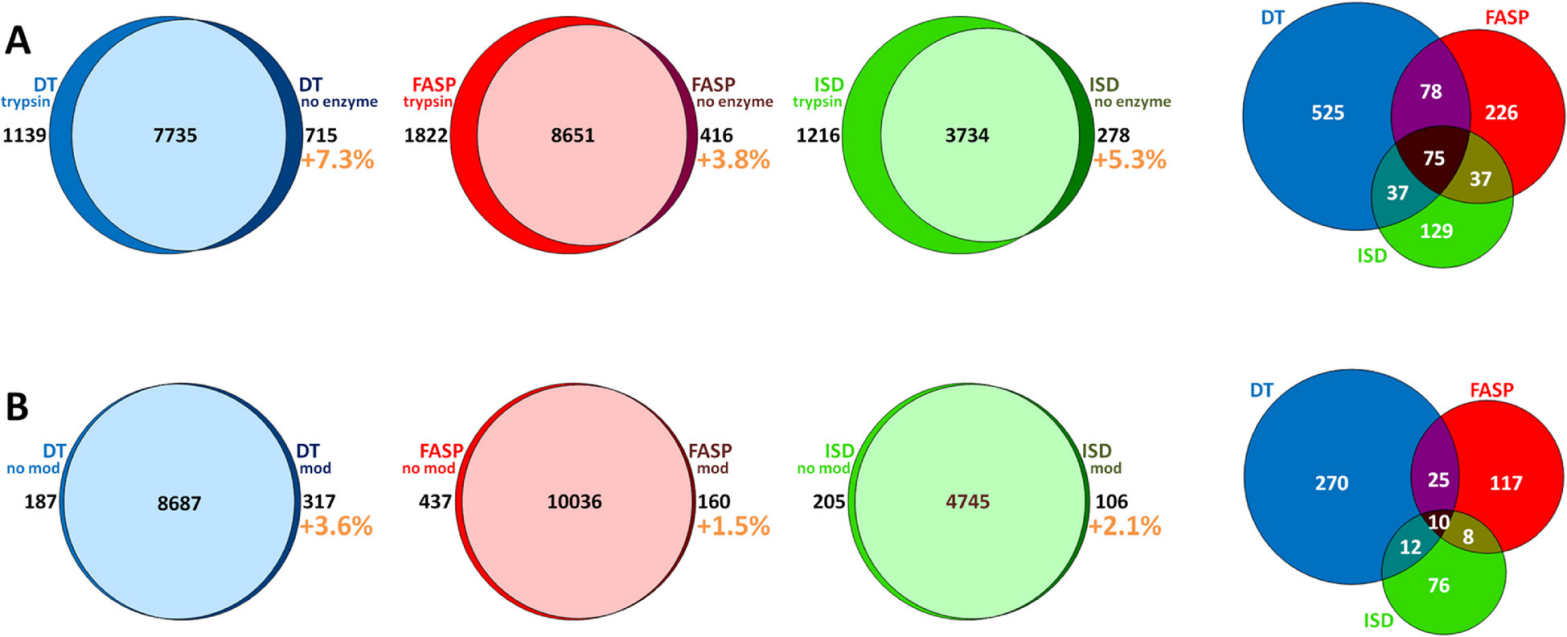
**Impact of non-tryptic and formaldehyde-modified peptides. A)** Left: Venn diagrams showing distribution of peptides identified with ‘trypsin’ and ‘no enzyme’ searches in DT (blue), FASP (red) and ISD (green) samples. Right: Venn diagram showing distribution of non-tryptic peptides among all methods. **B)** Left: Venn diagrams showing distribution of peptides identified with standard search (‘no mod’) and search comprising formaldehyde-induced modifications (‘mod’) in DT (blue), FASP (red) and ISD (green) samples. Right: Venn diagram showing distribution of formaldehyde-modified peptides among all methods.

MS spectra were further subjected to a “no enzyme” database search, in order to determine the percentage of non-tryptic peptides (NTPs) detectable with each method. The percentage increment in the number of peptide identifications achieved by performing the “no enzyme” search was found to be 7.3% for DT, 5.3% for ISD and 3.8% for FASP (Figure 5A, left). This additional search led to the identification of 1107 novel peptides in total, of which only 7% shared by all methods and 47% unique to DT (Figure 5A, right).

As a further investigation, a database search was performed by setting a previously described formaldehyde-induced mass shift (+12 Da) as variable modification on N-terminus, lysine, tryptophan and tyrosine [3,38]. This supplementary analysis enabled a 3.6% increase in the number of identified peptides for DT, versus 2.1% and 1.5% for ISD and FASP, respectively (Figure 5B, left). In absolute terms, nearly three times more putatively formaldehyde-modified peptides (FMPs) could be found in DT samples when compared to FASP and ISD; out of 518 FMPs identified in total, only 2% were common to all methods, while over 50% were unique to DT (Figure 5B, right). In detail, among the modifications detected, those involving the N-terminus were the most represented ones (56.4%), followed by those towards lysine (20.1%), tyrosine (16.7%) and tryptophan (6.8%). Concerning single methods, the percentage of modifications found on N-terminus ranged from 68.2% for ISD to 58.6% for FASP, those observed on lysine from 19.9% for DT to 10.3% for ISD, those detected on tyrosine from 18.7% for FASP to 12.2% for DT, and those measured on tryptophan from 6.4% for FASP to 4.6% for ISD.

Finally, no significant differences could be observed when increasing the maximum number of tryptic missed cleavages to 3 or 4, neither in general nor for a specific method.

## Discussion

The critical examination of the DT, FASP and ISD results allowed us to point out several typical advantages and drawbacks of each workflow, which appear to be consistently related to the technical features differing among the methods and to the nature of proteins after reaction with formaldehyde. Formalin fixation, in fact, causes a complex network of chemical bonds to form within the tissue molecules [39-41]. Since proteins undergo inter- and intramolecular crosslinks, they are thought to form a sort of “mesh” in which each protein forms a number of bonds proportional to the amount of formaldehyde-reactive residues (usually basic ones, especially lysine) which it contains [42-44]. Due to this reason, high-MW and basic proteins are known to be the most difficult to extract, separate and identify within an FFPE tissue proteome [5,6]. This leads to the hypothesis, clearly and consistently verified in this study, that sample preparation procedures based on a protein extraction step (such as FASP and ISD) may imply a depletion of high-MW proteins compared to methods founded on a whole tissue trypsinization. In fact, in the DT workflow the entire artificial “complex” of crosslinked proteins is directly digested, and tryptic peptides are expected to be released from all accessible proteins, without (or with lower) MW-related biases. On the other hand, data concerning protein pI (i.e., a lower mean pI in the DT proteome dataset compared to FASP and ISD) seem to be counterintuitive in respect to formalin-protein reactivity, since acidic proteins are expected to be relatively more abundant upon protein extraction than upon direct trypsinization. Possible explanations of this result might be a pI-related selectivity in protein solubilization due to the particular pH of the buffer used [45], or even the presence of low-MW, basic (and thus very reactive) proteins, widely linked to extracted proteins, whose peptides are released upon in solution or on filter trypsin digestion and contribute to increase the mean protein pI within the dataset [5].

Buffer characteristics, and especially their content in detergents, may also explain differences in membrane/hydrophobic proteins abundance among the three methods. Protein extraction (FASP and ISD) was indeed carried out using an SDS-based buffer (SDS was then depleted by replacement with urea and filter washes for FASP and by using detergent removal spin columns for ISD), whereas DT was performed with the commonly used ABC-based buffer (since SDS is generally not compatible with enzymatic digestion). We cannot exclude therefore that DT results could change if low amounts of SDS (trypsin activity is usually not impaired at SDS concentrations lower than 0.1%) or alternative labile or anionic detergents (e.g. RapiGest) are added to the DT buffer. Moreover, an increase of the number of hydrophobic peptides detected in ISD, when compared with FASP, was consistently described in a previous study [46].

It is also worth noting that, in this study, the detergent removal step included within the ISD protocol, carried out using spin columns, was performed on protein mixtures before trypsin digestion, while, according to several papers, similar columns were used to remove traces of detergent from peptide mixtures after digestion [24,46,47]. Previous papers have shown the successful application of an SDS depletion step on protein extracts before digestion, even leading to better results compared to FASP [48,49]. However, we cannot rule out that significant changes in the MS output could have been obtained if carrying out detergent removal after, and not before, trypsin digestion.

In addition, the three methods differ significantly with respect to some practical laboratory aspects. First, DT and ISD are slightly less labor-intensive when compared to FASP concerning experimental time and operator effort. Conversely, FASP produces very clean peptide mixtures, whilst issues in quantification for residual interefering substances occurred both for DT and ISD. Last, but not least, the DT workflow leads to a much lower extent of keratin contamination in comparison with FASP and ISD.

## Conclusions

In conclusion, the results presented in this study highlight that different sample preparation strategy can provide qualitatively and quantitatively different proteomic information, and that each method presents typical advantages and drawbacks that should be taken into account when planning a shotgun proteomic investigation dealing with FFPE tissue samples. A critical consideration of these data also advises that comparing results of FFPE tissue proteomic studies obtained using different methods might lead to incorrect and biased conclusions. On the contrary, in view of the considerable portion of unique identifications provided by each method (particularly by DT and FASP), a complementary, parallel use of different sample preparation strategies may be suggested, when a sufficient amount of tissue is available, in order to increase proteome coverage, width and depth.

## Methods

### FFPE tissue sample

Normal FFPE liver tissue was retrieved from the tissue archives of the Sassari University Hospital and de-identified. The tissue had been fixed in 10% neutral buffered formalin at ambient temperature for 36–48 h immediately after collection, and then processed for dehydration and paraffin embedding using standard procedures.

### Microtome slicing, deparaffinization and rehydration

After retrieval from the repository, the tissue block was sliced using a microtome, producing 45 serial FFPE tissue sections (5 μm thick), which were placed (in groups of 5) into 9 Eppendorf safe-lock tubes (Eppendorf, Hamburg, Germany) and weighed. Samples were deparaffinized and rehydrated according to a previously published procedure [50].

### Direct tissue trypsinization (DT)

Direct trypsinization of tissue samples was carried out based on a previously described protocol [18], with slight modifications. Tissues were homogenized in 50 mM ABC (100 μl buffer per 10 mg tissue) by harsh pipetting, incubated at 99°C for 60 min at 500 rpm in a Thermomixer Comfort (Eppendorf), reduced by incubating in 100 mM DTT, 50 mM ABC (50 μl buffer per 10 mg tissue) for 30 min at 56°C, alkylated by incubating in 100 mM iodoacetamide (IAM), 50 mM ABC (50 μl buffer per 10 mg tissue) for 20 min in the dark, and digested by incubating with trypsin diluted in 50 mM ABC (20 μg enzyme per 10 mg tissue) overnight at 37°C. The digestion was stopped by adding 10 μl of 20% trifluoroacetic acid (TFA). Samples were then centrifuged at 16,000 × g for 10 min and each peptide containing supernatant was collected, dried out and eventually reconstituted with 0.2% formic acid. Peptide concentration was estimated by measuring absorbance at 280 nm with a NanoDrop 2000 spectrophotometer (Thermo Scientific, San Jose, CA, USA), using dilutions of the MassPREP E. Coli Digest Standard (Waters, Milford, MA, USA) to generate a calibration curve.

### Protein extraction

Full-length protein extraction was carried out as described previously [50], with minor modifications. Briefly, tissue samples were homogenized in 20 mM Tris–HCl (pH 8.8), 2% SDS, 200 mM DTT (100 μl buffer per 10 mg tissue) by gentle pipetting and incubated at 99°C for 60 min at 500 rpm in a Thermomixer Comfort (Eppendorf). Samples were then centrifuged at 16,000 × g for 10 min and each protein containing supernatant was collected. Protein concentration was estimated using the EZQ quantification kit (Molecular Probes, Eugene, OR, USA).

### Filter-aided sample preparation (FASP)

Protein extracts were processed according to the “FASP II” protocol [36], modified as illustrated elsewhere [48]. Briefly, approximately 20 μg of protein extract were diluted tenfold in 8 M urea, loaded into the Microcon Ultracel YM-30 filtration devices (Millipore, Billerica, MA, USA), and centrifuged at 14,000 × g for 15 min. The concentrates were then diluted in 8 M urea and centrifuged again. After centrifugation, proteins were reduced in 10 mM DTT for 30 min, and then alkylated in 50 mM IAM for 20 min. After 5 washes (3 in 8 M urea and 2 in 50 mM ABC), trypsin solution was added to the filter (enzyme-to-protein ratio 1:100 w/w), and samples were incubated at 37°C overnight. Peptides were collected by centrifugation followed by an additional wash with an elution solution (70% ACN, 1% formic acid). Finally, the peptide mixture was brought to dryness and reconstituted in 0.2% formic acid to an approximate final concentration of 1 mg/ml. Peptide mixtures concentration was estimated by measuring absorbance at 280 nm as described above.

### In solution digestion (ISD)

Protein extracts were diluted with Milli-Q water to a final 0.2% SDS concentration, then dispensed on the top of Detergent Removal Spin Columns (Pierce, Rockford, IL, USA), incubated for 2 min at room temperature, and centrifuged for 2 min at 1,500 × g, according to the manufacturer’s instructions. The eluted solution was then sequentially incubated for 30 min at 56°C in 10 mM DTT, 50 mM ABC for reduction and for 30 min at room temperature in the dark in 25 mM IAM, 50 mM ABC for alkylation. Trypsin digestion was performed overnight at 37°C (enzyme-to-protein ratio 1:100 w/w). The digestion was stopped by adding 10 μl of 20% TFA and the solution was brought to dryness. Finally, the peptide mixture was resuspended in 0.2% formic acid to an approximate final concentration of 1 mg/ml.

### LC-MS/MS analysis

MS analysis was carried out using an LTQ-Orbitrap Velos (Thermo Scientific) interfaced with an UltiMate 3000 RSLCnano LC system (Dionex, Sunnyvale, CA, USA, now part of Thermo Scientific). Before loading, peptide mixtures were purified using ZipTip Pipette Tips (Millipore), according to the manufacturer’s recommendations. After loading, peptide mixtures (4 μg per run) were concentrated and desalted on a trapping pre-column (Acclaim PepMap C18, 75 μm × 2 cm nanoViper, 3 μm, 100 Å, Thermo Scientific), using 0.2% formic acid at a flow rate of 5 μl/min. The peptide separation was performed at 35°C using a C18 column (Acclaim PepMap RSLC C18, 75 μm × 15 cm nanoViper, 2 μm, 100 Å, Thermo Scientific) at a flow rate of 300 nL/min, using a 485 min gradient from 1 to 50% eluent B (0.2% formic acid in 95% ACN) in eluent A (0.2% formic acid in 5% ACN).

The mass spectrometer LTQ-Orbitrap Velos was set up in a data dependent MS/MS mode, as described previously [48]. Briefly, the lock mass option was enabled on a protonated polydimethylsiloxane background ion for internal recalibration, peptide ions were selected as the ten most intense peaks of the previous scan, and Higher Energy Collisional Dissociation (HCD) was chosen as the fragmentation method.

### Data analysis

Protein identification was performed using Proteome Discoverer (version 1.4.0.288; Thermo Scientific), with a workflow consisting of the following nodes (and respective parameters): Spectrum Selector for spectra pre-processing (precursor mass range: 350–5000 Da; S/N Threshold: 1.5), Sequest-HT as search engine (Protein Database: Homo sapiens sequences from UniProtKB/SwissProt, release 2013_12; Enzyme: Trypsin; Max. missed cleavage sites: 2; Peptide length range 5–50 amino acids; Max. Delta Cn: 0.05; Precursor mass tolerance: 10 ppm; Fragment mass tolerance: 0.02 Da; Static modification: cysteine carbamidomethylation; Dynamic modification: methionine oxidation), and Percolator for peptide validation (FDR < 1% based on peptide q-value). When necessary, “Enzyme” parameter was changed to “No Enzyme”, or four dynamic modifications were added corresponding to a +12 Da mass shift towards N-terminus, lysine, tryptophan and tyrosine.

Protein annotations concerning subcellular localization were retrieved from UniProtKB (http://www.uniprot.org), GRAVY scores were determined using the GRAVY Calculator (http://www.gravy-calculator.de), and transmembrane helices were predicted using the TMHMM Server (v.2.0, http://www.cbs.dtu.dk/services/TMHMM). The Normalized Spectral Abundance Factor (NSAF) was calculated according to Zybailov et al. in order to estimate protein and peptide abundance [51]; NSAF values were multiplied by 15,000 and approximated in order to deal with integers and facilitate comparisons. The NSAF log ratio was calculated as previously described [5] and used to estimate the extent of differential protein abundance. Unsupervised hierarchical clustering was carried out using Perseus (v.1.4.0.17, http://www.perseus-framework.org) using z-standardized NSAF data, after filtering out records without non-zero values in at least 2 replicates of at least one group and replacing missing values with values from the lower part of normal distribution (imputation width = 0.3, shift = 1.8), as shown elsewhere [31]. Statistical significance of differential protein abundance was determined using Perseus by applying Student’s t test (two-sample comparison, p < 0.05) to logarithmized (normally distributed) NSAF values. Quantitative data were parsed using in-house scripts, and graphs were generated using Microsoft Excel, Perseus and Venn Diagram Plotter (http://omics.pnl.gov/software/VennDiagramPlotter.php).

Mass spectrometry raw data, annotated spectra of the identified peptides, detailed search parameters and complete protein and peptide lists are available in the PeptideAtlas repository at http://www.peptideatlas.org/PASS/PASS00441.

## Abbreviations

ABC: Ammonium bicarbonate; ACN: Acetonitrile; DT: Direct tissue trypsinization; FASP: Filter-aided sample preparation; FFPE: Formalin-fixed paraffin-embedded; FMP: Formaldehyde-modified peptide; IAM: Iodoacetamide; ISD: In solution digestion; NSAF: Normalized spectral abundance factor; NTP: Non-tryptic peptide; PSM: Peptide-spectrum match; TFA: Trifluoroacetic acid; TMD: Transmembrane domain.

## Competing interests

The authors declare they have no competing interests.

## Authors’ contributions

AT conceived the study, performed sample preparation, analyzed the data and drafted the manuscript. MA contributed to sample preparation, data analysis and manuscript drafting. SP and DP carried out the mass spectrometry measurements. SU participated in study design and coordination. MFA participated in study design and coordination, data interpretation, and manuscript drafting. All authors read and approved the final manuscript.

## Supplementary Material

Additional file 1 Summary of protein, peptide and PSM identifications. Click here for file

Additional file 2 **Percentage protein distribution according to subcellular localization.** Mean and SD value of protein percentage for three independent experimental replicates are shown. Asterisks indicate statistical significance according to Student’s t-test (p value < 0.05); the blue ones indicate statistically significant difference versus DT, the red ones versus FASP, the green ones versus ISD and the black ones versus all other methods, respectively. Click here for file

Additional file 3 **Percentage protein distribution according to physicochemical features.** Percentage protein distribution according to MW (A), pI (B), number of transmembrane domains (TMD, C) and hydrophobicity (GRAVY score, D). Mean and SD value of protein percentage for three independent experimental replicates are shown. Asterisks indicate statistical significance according to Student’s t-test (p value < 0.05); the blue ones indicate statistically significant difference versus DT, the red ones versus FASP, the green ones versus ISD and the black ones versus all other methods, respectively. Click here for file

Additional file 4 Complete list of protein identifications, along with log ratio values to estimate differential expression between methods. Click here for file
