# Supplementary figures and images for: Critical comparison of sample preparation strategies for shotgun proteomic analysis of formalin-fixed, paraffin-embedded samples: insights from liver tissue
Source: Clin Proteomics. 2014 Jul 8;11(1):28. doi: 10.1186/1559-0275-11-28 (PMC4115481; doi:10.1186/1559-0275-11-28)

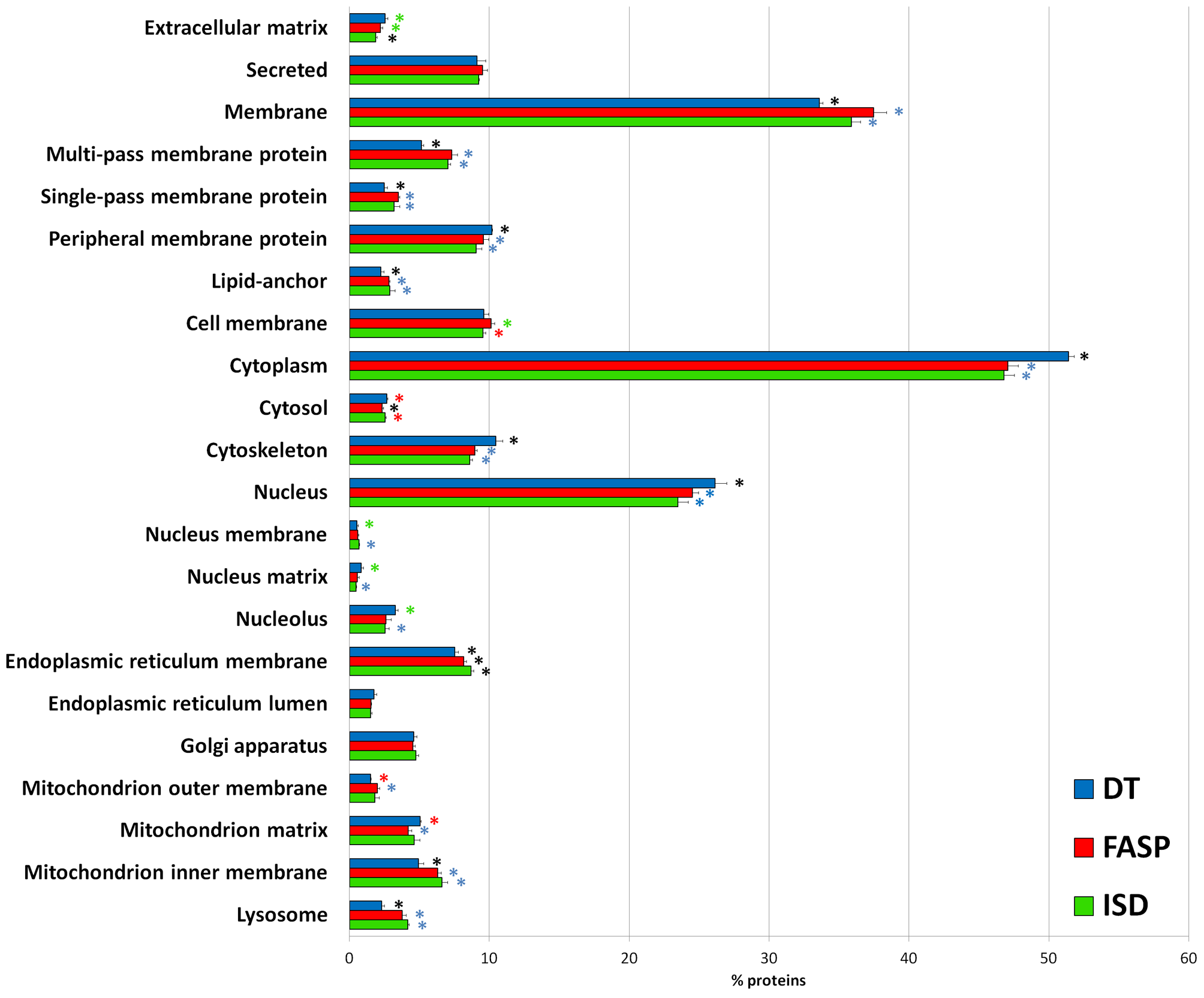

Supplement: Additional file 2 — Percentage protein distribution according to subcellular localization. Mean and SD value of protein percentage for three independent experimental replicates are shown. Asterisks indicate statistical significance according to Student’s t-test (p value < 0.05); the blue ones indicate statistically significant difference versus DT, the red ones versus FASP, the green ones versus ISD and the black ones versus all other methods, respectively. [file 1559-0275-11-28-S2.tiff]

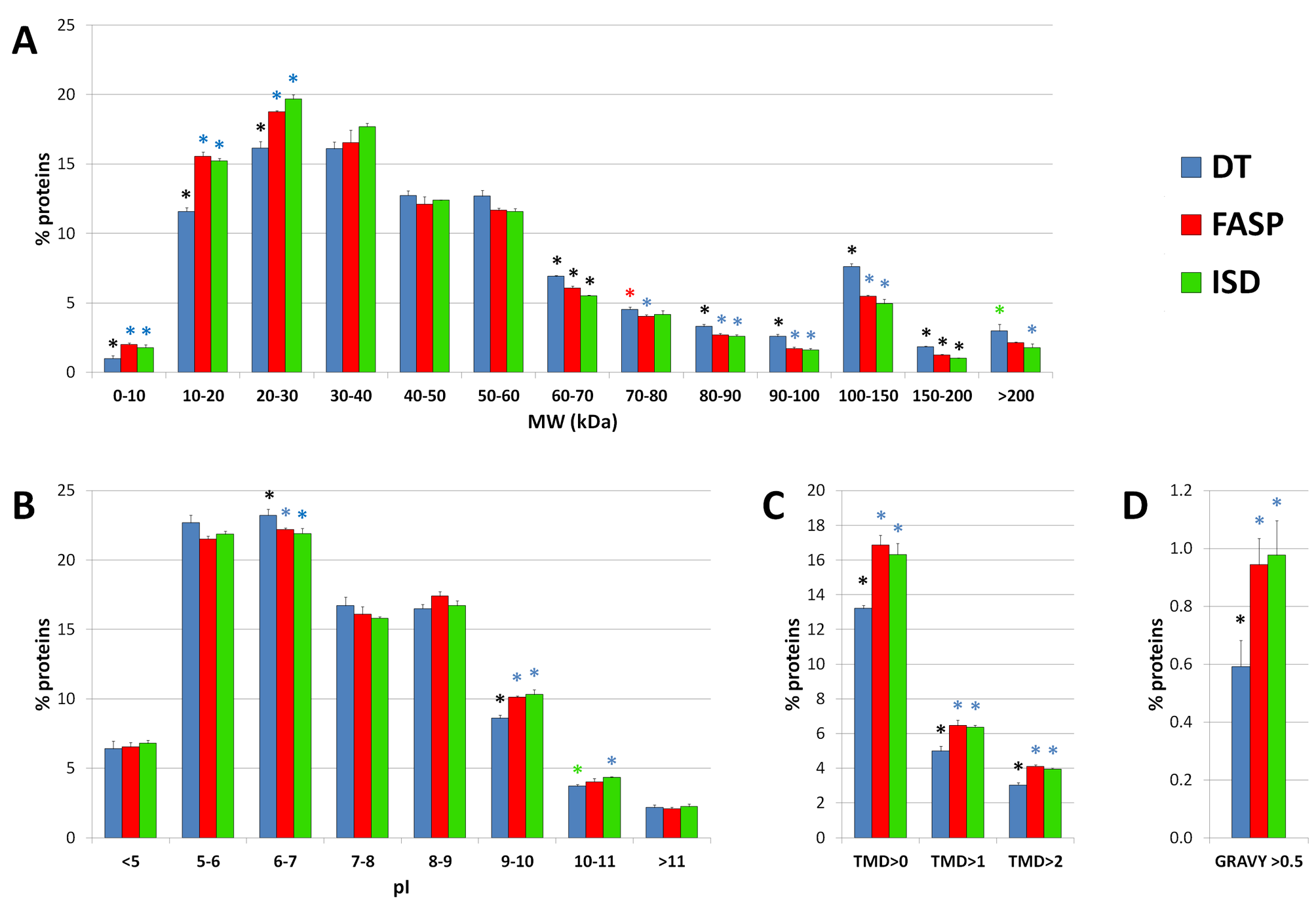

Supplement: Additional file 3 — Percentage protein distribution according to physicochemical features. Percentage protein distribution according to MW (A), pI (B), number of transmembrane domains (TMD, C) and hydrophobicity (GRAVY score, D). Mean and SD value of protein percentage for three independent experimental replicates are shown. Asterisks indicate statistical significance according to Student’s t-test (p value < 0.05); the blue ones indicate statistically significant difference versus DT, the red ones versus FASP, the green ones versus ISD and the black ones versus all other methods, respectively. [file 1559-0275-11-28-S3.tiff]
